# Kinetic studies of dexamethasone degradation in aqueous solution via a photocatalytic UV/H_2_O_2_/MgO process

**DOI:** 10.1038/s41598-022-25577-5

**Published:** 2022-12-09

**Authors:** Ghorban Asgari, Mehdi Salari, Mohammad Molla Mahmoudi, Reza Jamshidi, Ali Dehdar, Hossein Faraji, Solmaz Zabihollahi, Saber Alizadeh

**Affiliations:** 1 Department of Environmental Health Engineering, School of Public Health, Hamadan University of Medical Sciences, Hamadan, Iran; 2 Social Determinants of Health Research Center (SDHRC), Department of Environmental Health Engineering, School of Public Health, Hamadan University of Medical Science, Hamadan, Iran; 3 Department of Environmental Health Engineering, School of Health, Sabzevar University of Medical Sciences, Sabzevar, Iran; 4 Health Systems Research, Health Research Institute, Babol University of Medical Sciences, Babol, Iran; 5 Faculty of Chemistry, Bu-Ali-Sina University, Hamedan, Iran

**Keywords:** Environmental sciences, Environmental chemistry

## Abstract

Wastewaters discharged from different industries and hospitals may contain pharmaceuticals, especially dexamethasone (DEX). Thus, we applied the UV/H_2_O_2_ photocatalytic method in the presence of the MgO nanoparticles to remove dexamethasone from synthetic wastewater. Moreover, the effects of parameters such as pH (3–11), hydrogen peroxide concentration (1–8 mM), initial DEX concentration (5–30 mg/L), and catalyst dosage (0.01–0.2 g/L) during the reaction times (0–30 min) were investigated. Furthermore, the efficiency of UV/H_2_O_2_ in the presence and absence of catalysts was investigated. The photocatalyst is characterized by X-ray diffraction (XRD), field emission scanning electron microscope (FE-SEM), and Fourier-transform infrared spectroscopy (FTIR) techniques. It was found that the removal rate was enhanced by decreasing pH and the initial dexamethasone concentration. The removal rate was enhanced somewhat with concentrations of hydrogen peroxide and MgO. In the case of UV/H_2_O_2_/MgO, 87% removal efficiency was achieved, under the optimal conditions: pH 3, contact time of 30 min, dexamethasone concentration of 20 mg/L, H_2_O_2_ of 0.5 mM, and UV radiation of 55 watts. The kinetic data indicated that the reaction followed the second-order kinetic model. The results showed that the UV/H_2_O_2_ photochemical process can efficiently remove dexamethasone from aqueous in the presence of a MgO catalyst, and the mineralization efficiency was reached at about 98%.

## Introduction

Over recent decades, huge quantities of effluent containing various contaminants have been discharged into water bodies. Pharmaceutical compounds, which are considered a group of emerging contaminants, can enter the environment after or even without consumption^1^. Genetic mutations and sexual disorders may be due to the presence of these compounds in water^2^. Corticosteroids, which are widely applied to treat human and animal diseases, are a large class of drugs. They are entirely non-biodegradable and used to relieve inflammation in the human body. One of the most widely applied corticosteroids is dexamethasone (DEX) (Table 1). It is no wonder that wastewater produced by medical centers and hospitals contains a high content of this compound^3^.

**Table1:** The characteristics of dexamethasone.

Chemical formula	C_22_H_29_FO_5_
λ_max_	254 nm
Molar mass	392.461 g.mol^−1^
Chemical structure	

UV/MgO processes.

Many processes, like membrane bioreactors^4^, carbon nanotubes^5^, and anaerobic reactors^6^, have been utilized to treat wastewater containing pharmaceuticals. It should be noted that processes such as electrical coagulation^2^ and adsorption^7^ have been used for dexamethasone. Biological treatment methods are time-consuming and a high volume of sludge is generated in the electrical coagulation process^2^. Moreover, adsorption is not a cost-effective method because of the high cost of waste disposal. The basis of the advanced oxidation processes (AOPs) is the generation of hydroxyl radicals capable of degrading persistent organic pollutants (POPs)^8^. Also, these methods have attracted much attention owing to their capability in the treatment of a wide range of organic materials, which cannot be degraded by conventional chemical and biological methods^9^. In general, strong oxidants like hydrogen peroxide and ozone are generated or methods like ultrasonic, electron beam, and so forth are applied^10^. Among various AOPs, the application of photocatalytic processes is increasingly considered due to the possibility of producing cost-effective and efficient photocatalysts^11,12^. In a photocatalytic reaction, a catalyst is exposed to visible or UV light irradiation to generate hydroxyl radicals^13^. In photocatalytic processes, semiconductors like TiO_2_, ZnO, CdS and ZrO_2_ have mainly been used for organic matter degradation^14^. In general, nanoparticles are suitable for chemical reactions and the adsorption of different organic materials because of their high specific surface area^15^. Among these nanoparticles, MgO, which is a basic oxide, has various applications as a catalyst. Magnesium oxide (MgO) is a semiconductor whose unique chemical, mechanical, optical, and electrical properties, wide energy band gap, stability, inexpensive, and non-toxicity have made it very attractive for application photocatalytic processes^16^. It has been observed that the use of MgO nanoparticles in concert with catalytic ozonation can enhance the degradation of organic pollutants^17^. In this research, we tried to investigate the catalytic impact of MgO nanoparticles alongside UV radiation for hydrogen peroxide activation. Thus, in the presence of the MgO nanoparticles, the performance of the UV/H_2_O_2_ process in DEX removal from the aqueous environment was assessed.

## Materials and methods

### Chemicals and photoreactor

**Figure 1:**
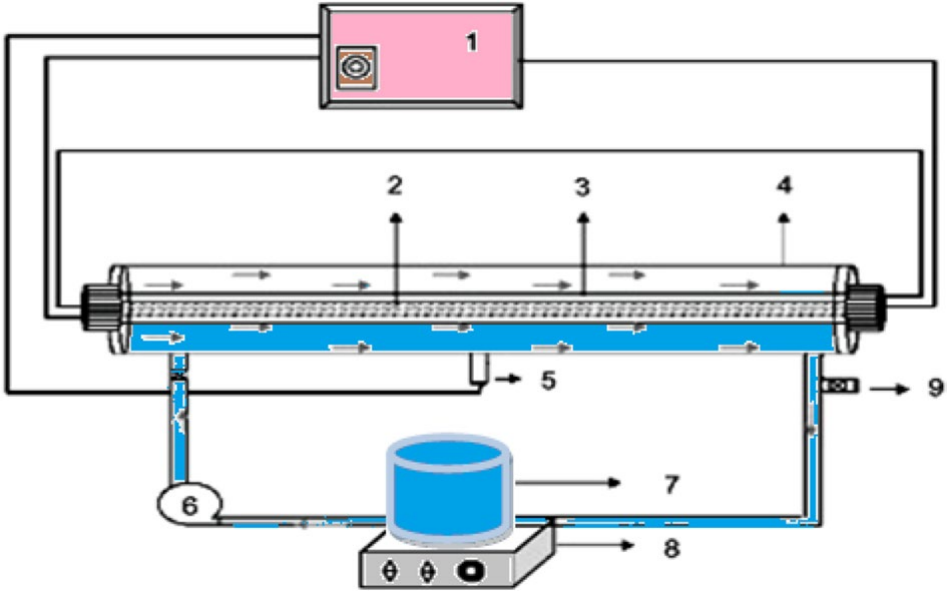
A schematic diagram of the reactor: (1) transformer, (2) low-pressure mercury-vapor UV lamp, (3) quartz cover (L: 85 cm D: 5 cm), (4) stainless steel box (L: 85 cm, D: 8 cm), (5) photocell, (6) pump (50–400 ml/min), (7) beaker (2 L), (8) shaker, and (9) sampling tube.

All the chemicals utilized in this study, H_2_O_2_, NaOH and H_2_SO_4_, and radical scavengers: ascorbic acid (AA), ethylenediamine tetraacetic acid (EDTA), and tert-butyl alcohol (TBA), were purchased from Sigma Aldrich and Merck Co., Germany. The MgO nanoparticle powder was purchased from ASPI Co. and sodium dexamethasone phosphate (C_22_H_28_FNa_2_O_8_P) was bought from Darou Pakhsh Pharmaceutical Co. For the tests, a 2-L stainless steel photoreactor, which was equipped with quartz glass and a 55-W low-pressure lamp (Philips Co.), was employed. A pump was used in order to continuously mix the samples. Figure 1 presents all the details of the reactor. To determine the dexamethasone degradation intermediates, liquid chromatography-mass spectrometry (LC–MS; Shimadzu LC/MS 2010 A) was used^18,19^.

### Experimental procedures

All experiments were performed in a batch-flow pilot. In the current research, at the fixed intensity of ultraviolet light, the impacts of a pH of 3–11, an initial DEX content of 5–30 mg/L, a hydrogen peroxide content of 1–8 mM, a MgO dose of 0.01–0.2 g/L, and a contact time of 0–30 min on the process performance were studied. All runs were carried out in triplicate at room temperature (25 °C), and average figures were recorded. Both first- and second-order linear kinetics were used to obtain the best-fitting model for the removal process. In this work, a solution containing 100 mg/L of the pollutant was prepared. Next, the working concentrations were prepared from this stock solution. A UV/Vis spectrophotometer (wavelength = 241 nm) (DR 5000, Hach Co., Germany) was employed to determine the contents of the DEX solution. Furthermore, as superoxide anion (O_2_^·–^), hole (h^+^), and hydroxyl radical (^·^OH), AA (0.2 mol/L), EDTA (0.2 mol/L), and TBA (0.2 mol/L) were used, respectively. In order to determine the mineralization rate of the pollutant during the process, the total organic carbon (TOC) of the experiments was detected using a Shimadzu 5000 TOC analyzer. Moreover, the chemical oxygen demand (COD) was determined according to the procedure expressed in Standard Methods (Federation and Association). A digital pH meter (Hach) was applied to determine the pH values. In the end, all the charts were plotted and the data was analyzed by means of Excel 2013. X-ray powder diffraction (XRD) was used to determine the crystal phases of the MgO nanoparticles (Rigaku Ultima IV). Field emission scanning electron microscopy (FE-SEM; FEI Nova NanoSEM 450) was used to examine the morphologies of the catalyst surface. Moreover, Fourier-transform infrared spectroscopy (FTIR) (Thermo, AVATAR) using a pellet generated by integrating the powder sample with KBr was used to observe functional groups of MgO nanoparticles.

## Results and discussion

### The characterization of the catalyst

**Figure 2:**
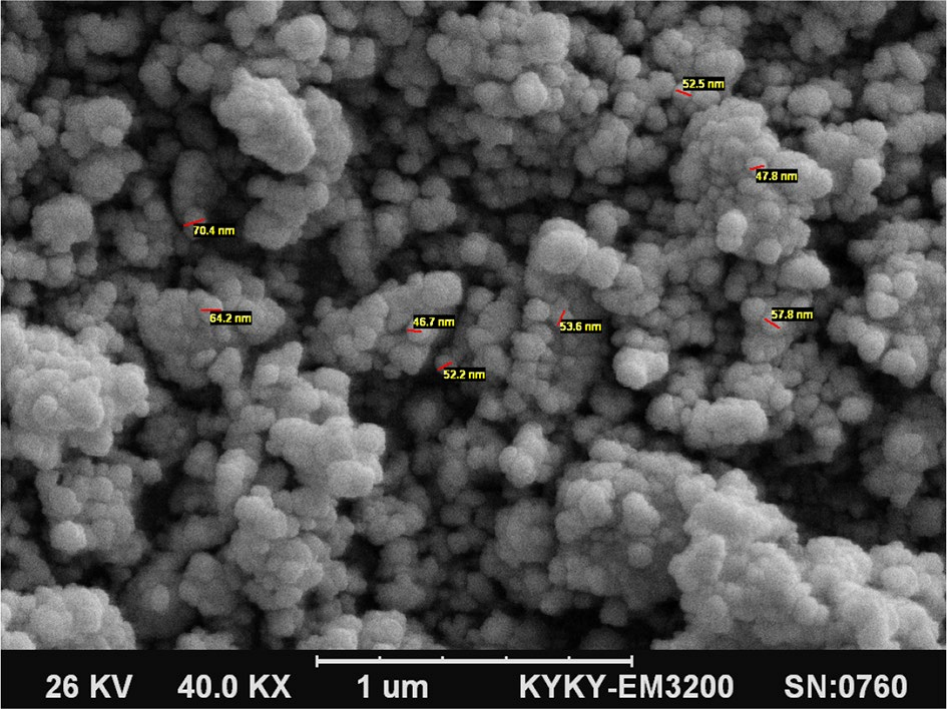
Scanning electron microscope (SEM) image of the MgO nanoparticles.

**Figure 3:**
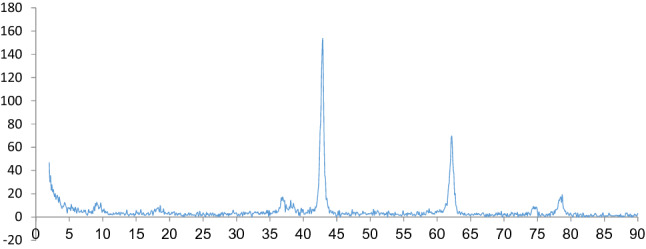
XRD analysis of the MgO nanoparticles.

**Figure 4:**
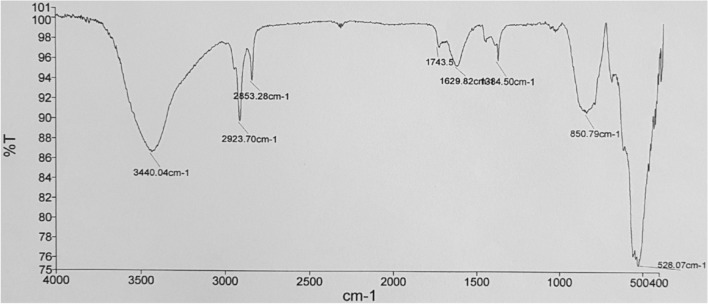
FTIR spectra of the MgO nanoparticles.

Figure 2 presents the morphological properties of the nanoparticles as determined by the scanning electron microscopy (SEM) analysis, performed before starting the reaction. As can be seen, it is obvious that the magnesium oxide nanoparticles had a porous, spongy structure. Figure 3 illustrates, in the amorphous, shape, two peaks seen in 2θ = 43 and 62, illustrating the presence of cubic MgO, and the peaks can be assigned to a pure phase of MgO. The FTIR spectrum was also tested for investigation and identification of the catalyst surface’s functional groups. It is well known that MgO chemisorbs H_2_O and CO_2_ molecules from the atmosphere due to its surface acid–base properties^20^. The major peaks appearing in the FT-IR spectra may be assigned to the following modes: (i) a broad vibration band around 2800–3700 cm^−1^ can be associated to OH stretching vibrations of the surface-bonded (or) adsorbed water, which was introduced in precursor solution (ii) the peak around 1629 cm^−1^ is devoted to OH bending vibrations of water molecules. According to Fig. 4, a strong peak band was detected at a wavelength of 528 cm^−1^, due to asymmetric vibrations of the Mg–O band. Peaks observed at 850 cm^−1^ corresponded to C=O stretching vibrations. (Comparative Study of Microwave and Conventional Methods for the Preparation and Optical Properties of Novel MgO-Micro and Nano-Structures) (MgO Nanoparticles Prepared By Microwave-Irradiation Technique and Its Seed Germination Application). The surface hydroxyl groups have been recognized to play an important role in the photocatalytic reaction since they can inhibit the recombination of photogenerated charge carriers, and also interact with the photogenerated holes to produce active oxygen species^20^.

### Impact of initial pH

**Figure 5:**
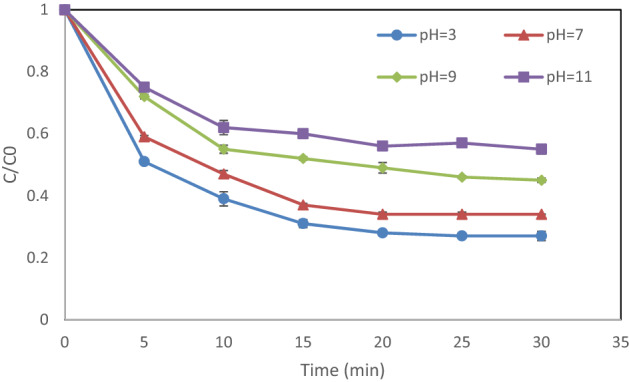
Impact of pH on DEX degradation: hydrogen peroxide dosage = 1 mM, DEX content = 20 mg/L, and MgO dosage = 0.05 g/L).

The literature review revealed that AOPs are completely pH-dependent^21^. Hence, in this study, at the fixed hydrogen peroxide content of 1 mM, the pH values were changed from 3 to 11 to investigate the changes in the removal efficiency. The maximum removal efficiency (73%) by the UV/H_2_O_2_/MgO method was attained at a pH of 3 (Fig. 5). These findings are attributed to the surface properties of the adsorbent and the ionization/degradation of the adsorbate. The number of hydrogen ion increases gradually with decreasing pH. When H^+^ is adsorbed, the positive charge on the nanoparticle’s surface increases and, in turn, the electrostatic force between the cationic charge on the surface of the nanoparticle and the negative DEX molecule enhances, increasing the adsorption rate. It was found that the performance declined sharply when pH was raised. For example, a 45% decrease was seen in removal efficiency at a pH value of 11 within 30 min. As can be seen, the degradation rate remained unchanged after 20 min and was insignificant after 30 min. Therefore, reaction times between 0 and 30 min were selected for the rest of the experiments. Furthermore, a decrease in the removal efficiency of the UV/H_2_O_2_ in alkaline conditions can be caused by a reaction between H_2_O_2_ and solution alkalinity; this causes hydroxyl radicals to go down. Moreover, in comparison with a neutral pH, the nanoparticles are accumulated in acidic conditions; as a result, the catalyst’s effective surface area was enhanced^22^.

### Impact of H_2_O_2_ dosage

**Figure 6:**
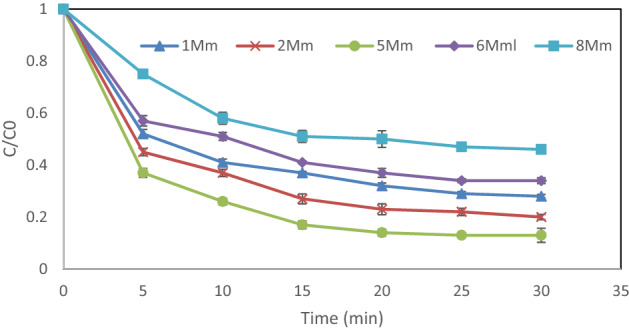
Impact of H_2_O_2_ dosage on the removal efficiency of DEX under the following conditions: pH = 3, DEX concentration = 20 mg/L and MgO dosage = 0.05 g/L.

In this study, under the following conditions: pH 3, DEX content of 20 mg/L, and catalyst dosage of 0.05 g/L, different initial contents of hydrogen peroxide (1–8 mM) were tested. According to the results presented in Fig. 6, the removal efficiency increased to 87% when the concentration of H_2_O_2_ was raised to 5 mM. It should be noted that, when the H_2_O_2_ concentration exceeded 5 mM, the removal efficiency started to decline. An excessive increase in H_2_O_2_ concentration causes part of ·OH to be inhibited and then HO_2_ is produced, which has a lower oxidation potential than ·OH (Eq. (1))^23^. Also, this decrease in performance can be because of the continuous degradation of H_2_O_2_ into oxygen and water as shown in Eq. (1)^24^.

1 $${\text{H}}_{{2}} {\text{O}}_{{2}} + \cdot{\text{OH}} \to {\text{ H}}_{{2}} {\text{O }} + {\text{ HO}}_{{2}}\cdot$$

### Impact of initial DEX concentration

**Figure 7:**
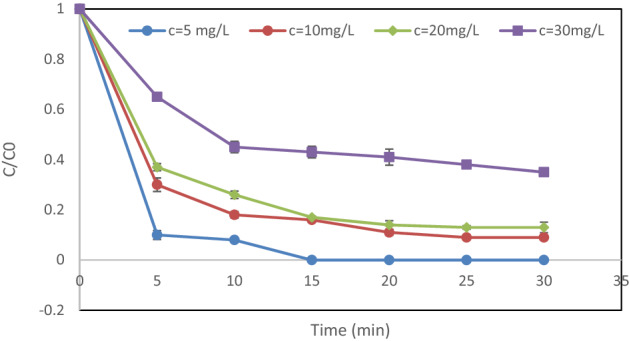
Impact of initial DEX content on DEX removal rate: pH = 3, hydrogen peroxide dose = 5 mM and MgO dose = 0.05 g/L.

In photocatalytic processes, how the initial concentration of the pollutant affects the removal efficiency is of great importance. Figure 7 shows the impact of the initial DEX content on the removal efficiency in UV/H_2_O_2_/MgO. As can be seen, with an increased DEX concentration from 5 to 30 mg/L, the removal efficiency declined. And, 65% of DEX was degraded at a concentration of 30 mg/L. Within 5 min of the reaction and an initial DEX content of 5 mg/L, a 90% removal efficiency was reached (Fig. 7). The decrease in the removal rate by increasing the concentration of DEX can be attributed to the fact that at all concentrations, the amount of nanoparticles, contact time, and pH are the same. As a result, the amount of radicals produced is similar at all concentrations. Naturally, it is expected to see lower DEX degradation at lower concentrations. By contrast, at a lower initial concentration, the number of active sites on the catalyst’s surface capable of degrading DEX increases. Furthermore, ultraviolet light cannot penetrate effectively into the solution when there are higher concentrations of DEX^25^.

### Impact of the dose of MgO

**Figure 8:**
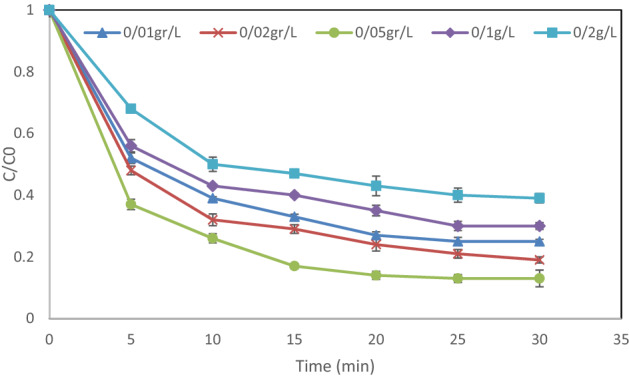
Impact of MgO dose on the rate of DEX removal: initial dexamethasone content = 20 mg/L, pH = 3, and hydrogen peroxide dosage = 5 mM.

In Fig. 8, it is shown how the changes in magnesium oxide (0.01–0.2 g/L) affected the removal efficiency of the pollutant in photo-oxidation. As can be seen, the removal efficiency went up with the increase in the dose of MgO. Nevertheless, when the dosage exceeded 0.05 g/L, the removal rate declined. At higher doses, there are more active sites and free electrons in the conductor, resulting in the generation of more hydroxyl radicals that can take part in the degradation^26^. Also, the removal rate of DEX at higher dosages of this nanoparticle was marginal, because the nanoparticles stuck together, causing the intensity of the UV lamp to decrease. Sobana et al. reported that during the photocatalytic reactions, the removal efficiency of Red Direct 23 increased as an increase in the number of active sites, resulting from a rise in the dosage of titanium dioxide doped with silver^27^. It should be noted that the current study’s findings are consistent with those of other related studies^16^.

### Impact of radical scavengers

**Figure 9:**
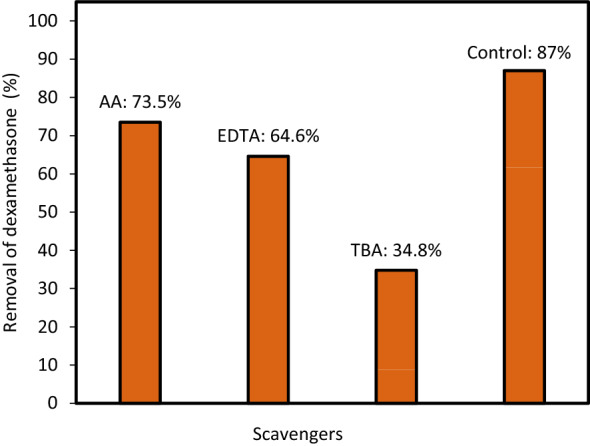
The degradation rate of DEX in the presence of different radical scavengers (initial DEX concentration = 20 mg/L, pH = 3, H_2_O_2_ dose = 5 mM, and MgO dose = 0.05 g/L).

In this study, the main reactive species in DEX degradation were identified using radical scavenging experiments under optimal conditions. To investigate the effects of different scavengers on DEX degradation, AA (0.2 mol/L), EDTA (0.2 mol/L), and TBA (0.2 mol/L) were added to the DEX solution as superoxide anion (O_2_^·−^), hole (h^+^), and hydroxyl radical (·OH) scavengers, respectively^28^. The results show three types of inhibition, corresponding to the three active species in the UV/H_2_O_2_/MgO process. From Fig. 9 it can be see that 87% of DEX can be removed in 30 min without a scavenger (Control). However, with the addition of AA, EDTA, and TBA, DEX removal efficiency decreased to 73.5%, 64.6% and 34.8%, respectively (Fig. 9). The rate of DEX degradation during the reaction process was less affected by the addition of AA (a scavenger of O_2_^·−^). Since TBA is a known ·OH scavenger^29^, the DEX degradation in the established UV/H_2_O_2_/MgO system in the presence of TBA clearly shows that the reaction with ·OH was the predominant active specie contributing to DEX removal. Furthermore, the decrease in DEX degradation in the presence of EDTA as a hole scavenger confirms h^+^ photogeneration. The hole reactive species directly or indirectly oxidizes DEX compounds by generating hydroxyl radicals through the oxidation of water molecules. Therefore, the main mechanism was discovered to be in the form of ·OH-driven reactions, confirming those ·OH radicals were key species in the UV/H_2_O_2_/MgO process in DEX degradation, as described in the following Eqs. (2)–(6)^28,30^:

2 $${\text{MgO }} + {\text{ light }} \to {\text{ e}}^{ - } + {\text{ h}}^{ + } ,$$

3 $${\text{h}}^{ + } + {\text{ H}}_{{2}} {\text{O }} \to \cdot {\text{OH }} + {\text{ OH}}^{ + } ,$$

4 $${\text{e}}^{ - } + \, 0.{\text{5 O}}_{{2}} + {\text{ H}}_{{2}} {\text{O }} \to \, \cdot {\text{OH }} + {\text{ OH}}^{ - } ,$$

5 $${\text{h}}^{ + } + {\text{ OH}}^{ - } \to \cdot {\text{OH}},$$

6 $${\text{DEX }} + \cdot {\text{OH}} \to {\text{Degradation Products}}.$$

This result corresponds with Akbari et al.^30^ study that stated hydroxyl radicals are the main mechanism in ciprofloxacin antibiotic removal using S, N-doped MgO nanoparticles under UVA-LED.

### TOC analysis and mineralization

**Figure 10:**
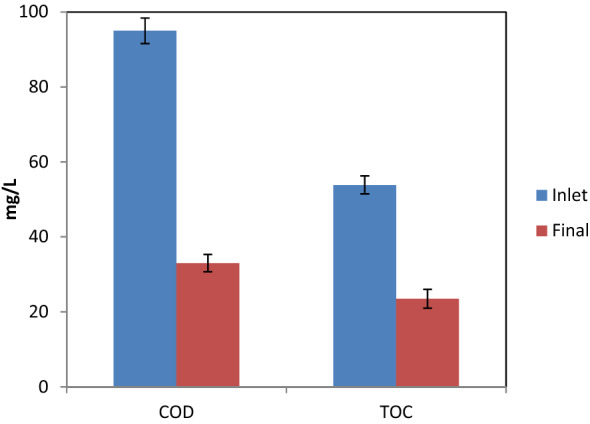
TOC and COD removal (initial DEX concentration = 20 mg/L, pH = 3, H_2_O_2_ dose = 5 mM, andMgO dose = 0.05 g/L).

In this study, the content of TOC was determined because DEX is initially converted to other degradation byproducts that are still organic. Thus, we determined the mineralization of DEX by recording TOC concentrations during the process. The TOC and COD concentrations of the samples were determined under the selected conditions (Fig. 10). It was found that the initial TOC was determined at 53.8 mg/L, and it declined to 23.5 mg/L after the exertion of the UV/H_2_O_2_/MgO process for 30 min, illustrating a mineralization rate of 56%. Accordingly, COD was reduced by up to 65%. However, at the same time of contact, the rate of DEX removal was 87%. Thus, it is claimed that for more mineralization, more contact time is required. For instance, the TOC removal rate increased to 98% within 120 min. It should be pointed out that lower by-products can be generated when a suitable contact time is regarded for reaching the mineralization rate of interest by the UV/H_2_O_2_/MgO process. It should be noted that, in the application of photocatalytic reactions, intermediates must be detected and eco-toxicological examinations should be performed.

### Comparison of the processes

**Figure 11:**
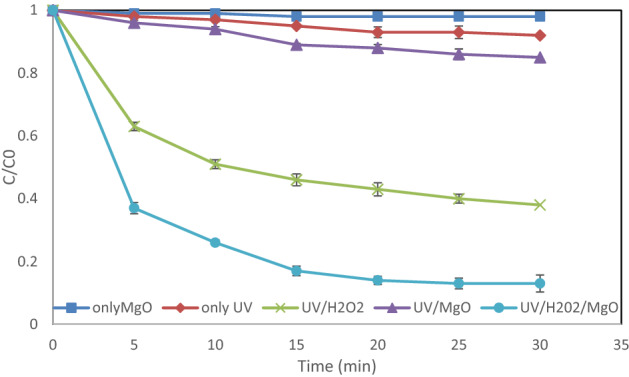
Comparison of the UV, UV/H_2_O_2_, UV/MgO and UV/H_2_O_2_/MgO processes under the optimum conditions.

In this study, the UV/H_2_O_2_ process was run in the presence and absence of the MgO catalyst. Also, the results of the UV and UV/MgO processes were compared. As indicated in Fig. 11, only 8% of the pollutant was degraded via the UV application within 30 min. Moreover, the performance of the UV/MgO process was nearly 17%, which may be because of the low adsorption rate that occurred on the surface of magnesium oxide. It should be noted that there was a dramatic difference between the removal efficiency rates of the UV/H_2_O_2_ photo-oxidation and the UV/H_2_O_2_/MgO process, which were found to be 61% and 87%, respectively. The activity of magnesium oxide in catalyzing oxidation decay was relative to the surface acid–base properties of the oxide. Water molecules can be adsorbed on the magnesium oxide’s surface due to the unsaturated state of surface electrons. As a result, surface hydroxyl groups may be formed. These groups play a basic role in the acid–base characterizations of magnesium oxide. Therefore, the process can be catalyzed well due to the surface hydroxyl groups. Thus, it is expected to see more DEX removal in the presence of magnesium oxide.

### Investigation of process kinetics

The behavior of DEX removal was studied by both the linear forms of pseudo-first and second-order kinetic models^31^ as expressed in Eqs. (7) and (8).

7 $${ln}_{Ct}={ln}_{C0}\times {e}^{-kt},$$

8 $$\frac{1}{{ln}_{Ct}}=\frac{1}{{ln}_{C0}}+{k}_{2}t.$$

**Figure 12:**
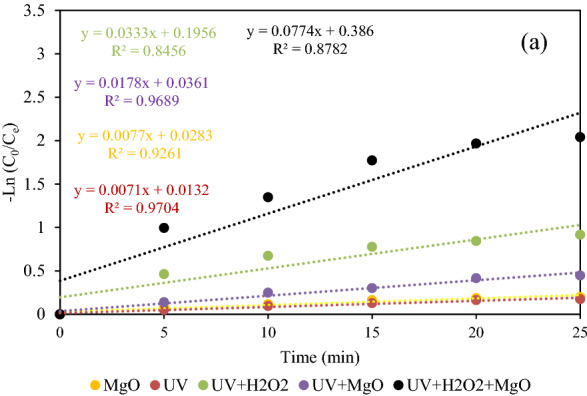
The degradation of DEX under MgO, UV, UV/H_2_O_2_, UV/MgO, and UV/H_2_O_2_/MgO processes based on a pseudo-first-order model.

**Figure 13:**
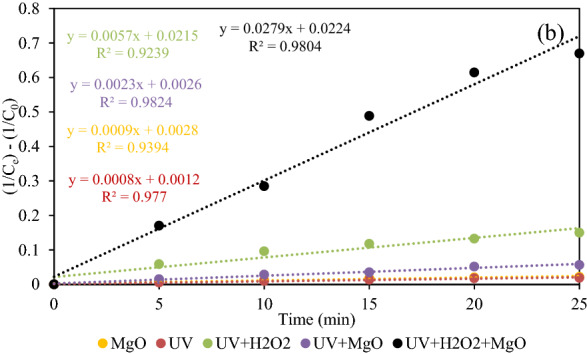
The degradation of DEX under MgO, UV, UV/H_2_O_2_, UV/MgO, and UV/H_2_O_2_/MgO processes based on a pseudo-second-order model.

Here, C_0_ and C_t_ show DEX concentration at times 0 and t (min), respectively. k_1_ (min^−1^) and k_2_ (mg/L.min) are assigned to the first and second-order kinetic constants, respectively. Figures 12 and 13 show pseudo-first and second-order kinetic models obtained by plotting Ln (c_t_/c_0_) and 1/c_t_–1/c_0_ against reaction time. The values of k_1_ and k_2_ obtained by the corresponding kinetic models are given in Table 2. In addition, the R^2^ values for all the single, binary, and ternary processes are better fitted to the pseudo-second-order kinetic model. The findings strongly indicate that the reaction constant for the UV/H_2_O_2_/MgO process was the highest among other methods of DEX removal. This illustrates that the combined UV/H_2_O_2_/MgO methods were more effective in DEX removal than MgO, UV, UV/H_2_O_2_, UV/MgO processes.

**Table 2:** The obtained coefficients of first and second order kinetic models for the removal of DEX by UV, MgO, UV/H_2_O_2_ and UV/H_2_O_2_/MgO processes were calculated.

Process	UV		MgO		UV/MgO		UV/H_2_O_2_		UV/H_2_O_2_/MgO	
	R^2^	K	R^2^	K	R^2^	K	R^2^	K	R^2^	K
First-order model	0.9704	0.0071	0.9261	0.0077	0.9689	0.0178	0.8456	0.0333	0.8782	0.0774
Second–order model	0.977	0.0008	0.9394	0.0009	0.9824	0.0023	0.9239	0.0057	0.9804	0.0279

### Degradation pathway of DEX

**Figure 14:**
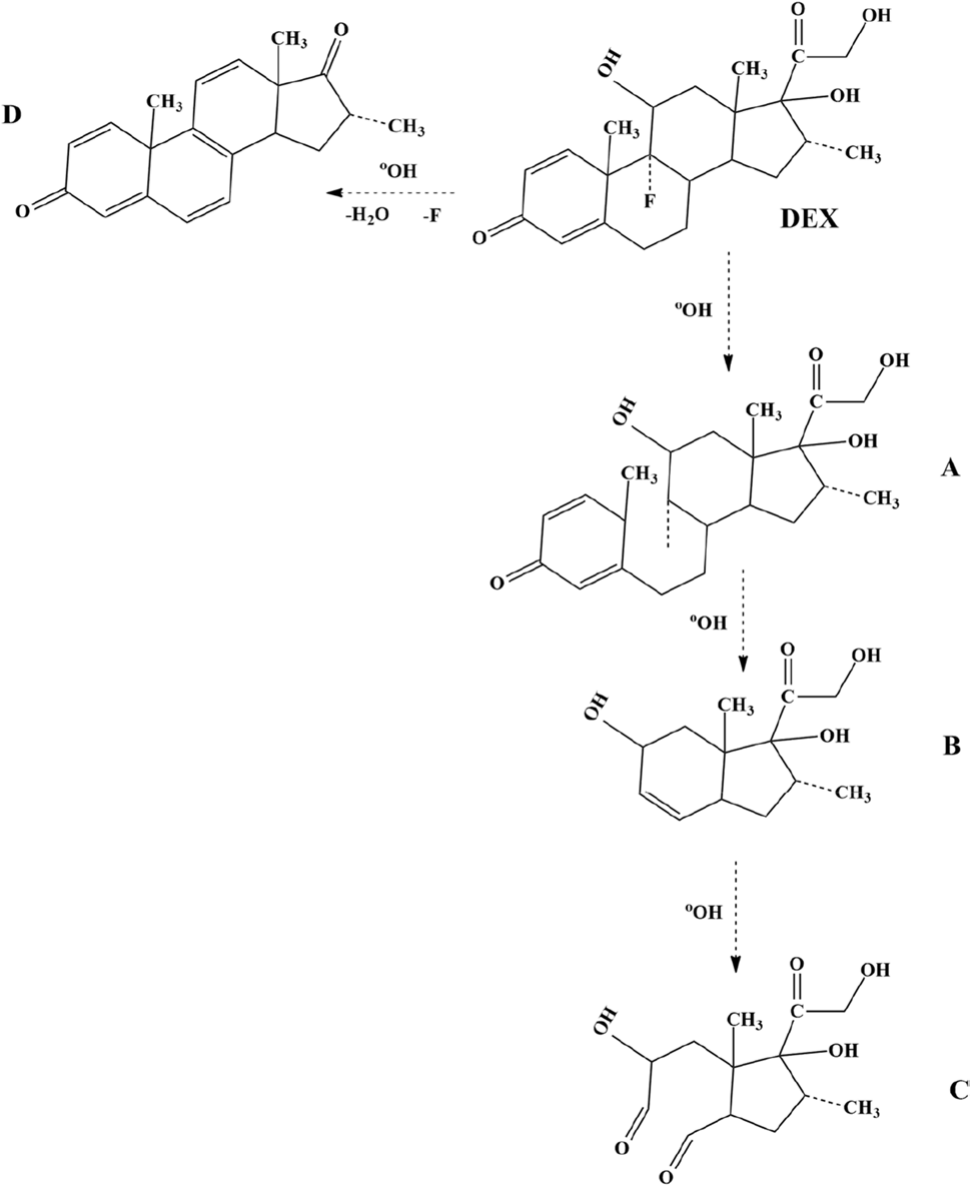
The degradation pathway of DEX under UV/H_2_O_2_/MgO process.

The hydroxyl radical interacts with organic pollutants quickly and strengthens C-unsaturated bonds. Because DEX molecules contain many OH groups, they are unstable to oxidation with a ·OH radical ^32^. In the present study, the intermediates were determined using the LC–MS method to determine the degradation pathway of DEX in the UV/H_2_O_2_/MgO process. Based on degradation intermediates of DEX and previously published data^32,33^, two possible pathways of DEX degradation were proposed (Fig. 14). In the first pathway, the attack ·OH radical to DEX molecule causes create a new intermediate (A). According to the intermediate (A), the products of (B), (C), and (D) could be attained by the cleavages of the bonds, which was related to the direct degradation of DEX compounds via the process of photodegradation. In the second pathway, the hydroxyl radical attack on the methylene group results in the mineralization of two carbon atoms and the creation of a ketone group on the remaining structure. The preferred loss and degradation in DEX are HF, which are continued by the combined losses of the HF and H_2_O molecules. (Intermediate D)^34^. Two water molecules are released after the ring breaks up. On further attack by the hydroxyl radical and after the breaking of the benzene rings, DEX was degraded to less refractory intermediate compounds, and thereby these compounds mineralized into CO_2_ and H_2_O^18^.

## Conclusion

In this study, dexamethasone was degraded by using H_2_O_2_ and hydroxyl radical generation-based AOP processes. It was found that, with decreasing pH and initial DEX concentration, the removal efficiency of the UV/H_2_O_2_/MgO method improved. Also, the following values were determined to be the optimum conditions: hydroxyl and magnesium oxide nanoparticle concentrations up to 5 mM and 0.05 g/L, an initial concentration of 20 mg/L, and a contact time of 30 min. The kinetic response illustrated that the obtained data followed the pseudo-first-order kinetic model. The findings also indicated that the UV/H_2_O_2_ method could dramatically degrade the pollutant from an aqueous solution when the MgO catalyst was applied as a catalyst (mineralization rate of 98%). Further, the catalytic activity of magnesium oxide is attributed to the surface acid–base characterization of this oxide. Finally, the used process can be considered a suitable method for the removal of pharmaceuticals under optimum conditions.

## Data Availability

All data generated or analyzed during this study are included in this published article. The datasets generated and/or analysed during the current study are available in the [Chemistry and Chemical biology] repository, [https://www.springernature.com/gp/authors/research-data-policy/repositories-chem/12327084]”.
